# Supplementary material for: Early Differentiation Signatures in Human Induced Pluripotent Stem Cells Determined by Non-Targeted Metabolomics Analysis
Source: Metabolites. 2023 May 29;13(6):706. doi: 10.3390/metabo13060706 (PMC10301689; doi:10.3390/metabo13060706)
Supplement: Supplementary file 1 [file metabolites-13-00706-s001.zip › Figures S1-S4.pdf]

Supplementary information

Early Differentiation Signatures in Human Induced Pluripotent Stem Cells Determined by Non-Targeted Metabolomics Analysis

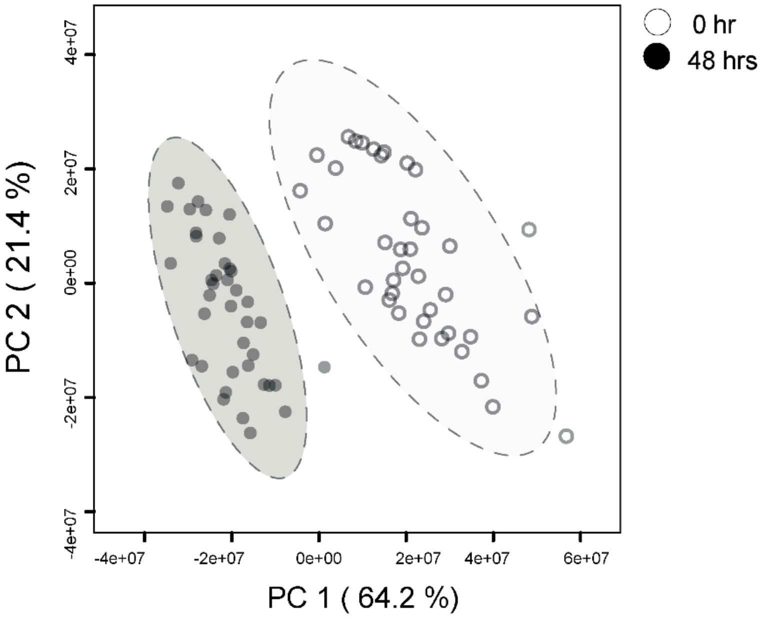

Figure S1. Principal component analysis (PCA) of metabolomics dataset peak areas. Analysis of samples under E6 alone as well as in combination with additives (T1 to T8) at 0 h and 48 h.

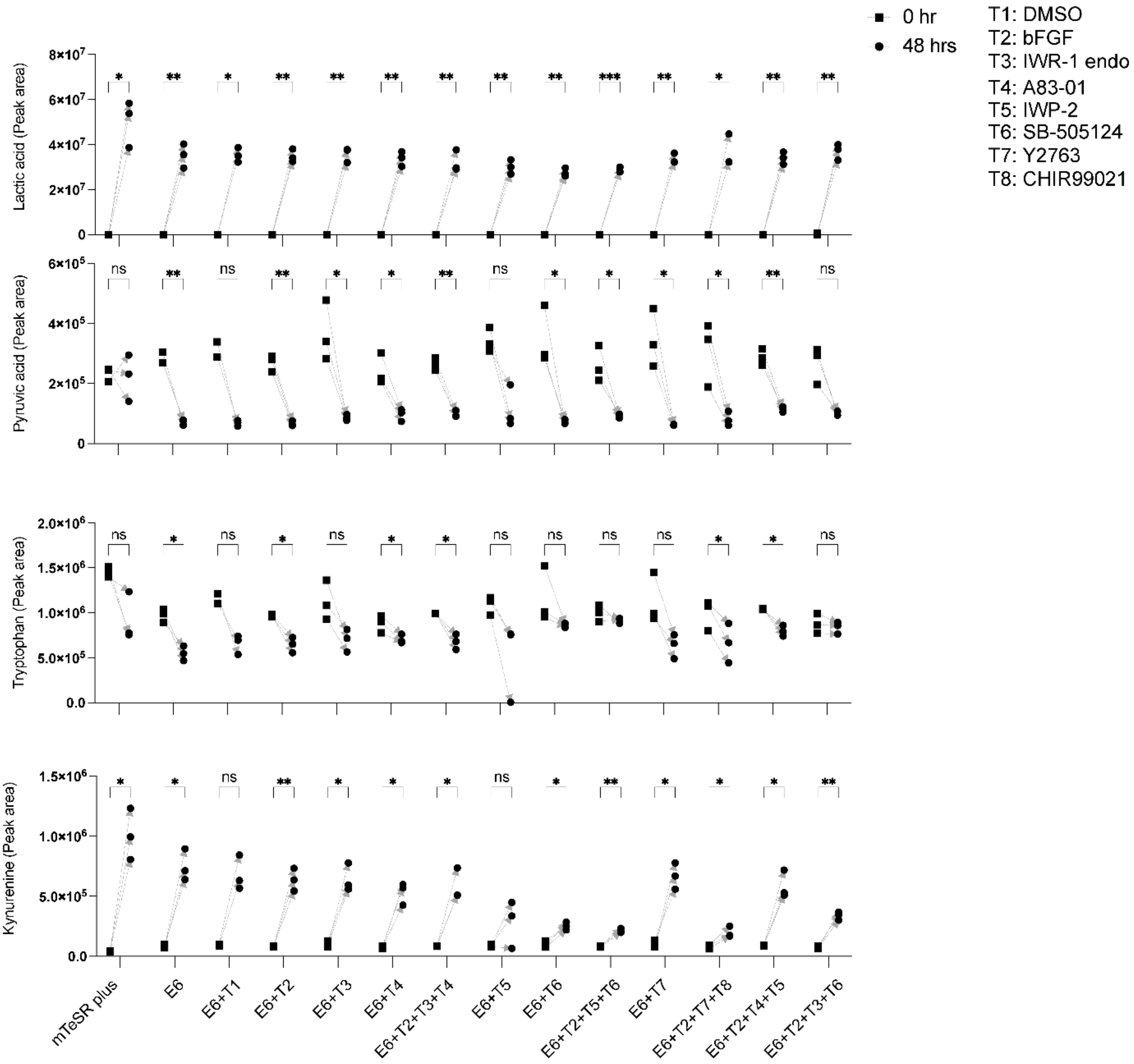

Figure S2. Analysis of extracellular metabolites from the human iPSC-585A1 at 0 h and 48 h. Representative examples of selected metabolites including: lactic acid, pyruvic acid, tryptophan, and kynurenine. The *p*-values were determined using a paired t-test, with a significance level of * $p < 0.05$, ** $p < 0.01$ and *** $p < 0.001$. No significant difference was noted for metabolites with a *p*-value of "ns".

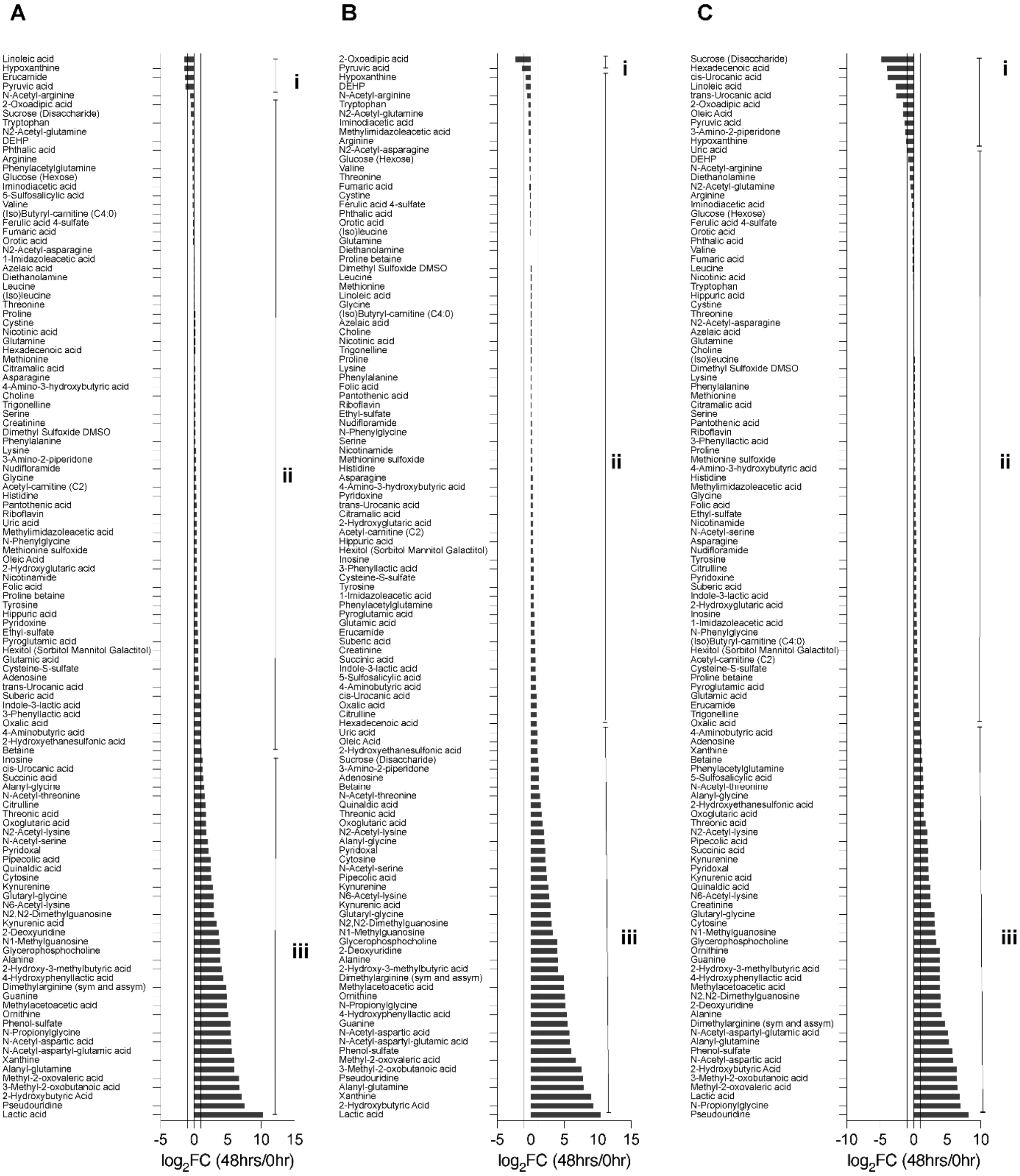

Figure S3. The determination of metabolites changes after 48 h. The ratio folds change ($\log_2$ 48 h/0 h) of metabolites abundances. (**A**) E6 in combination with A83-01. (**B**) E6 in combination with IWP-2_A83-01_bFGF. (**C**) E6 in combination with IWR-1 endo _ SB505124_ bFGF. Category i (log2 fold change ≤ −1), Category ii (log2 > −1 and log2 < 1), and Category iii (log2 fold change ≥ 1).

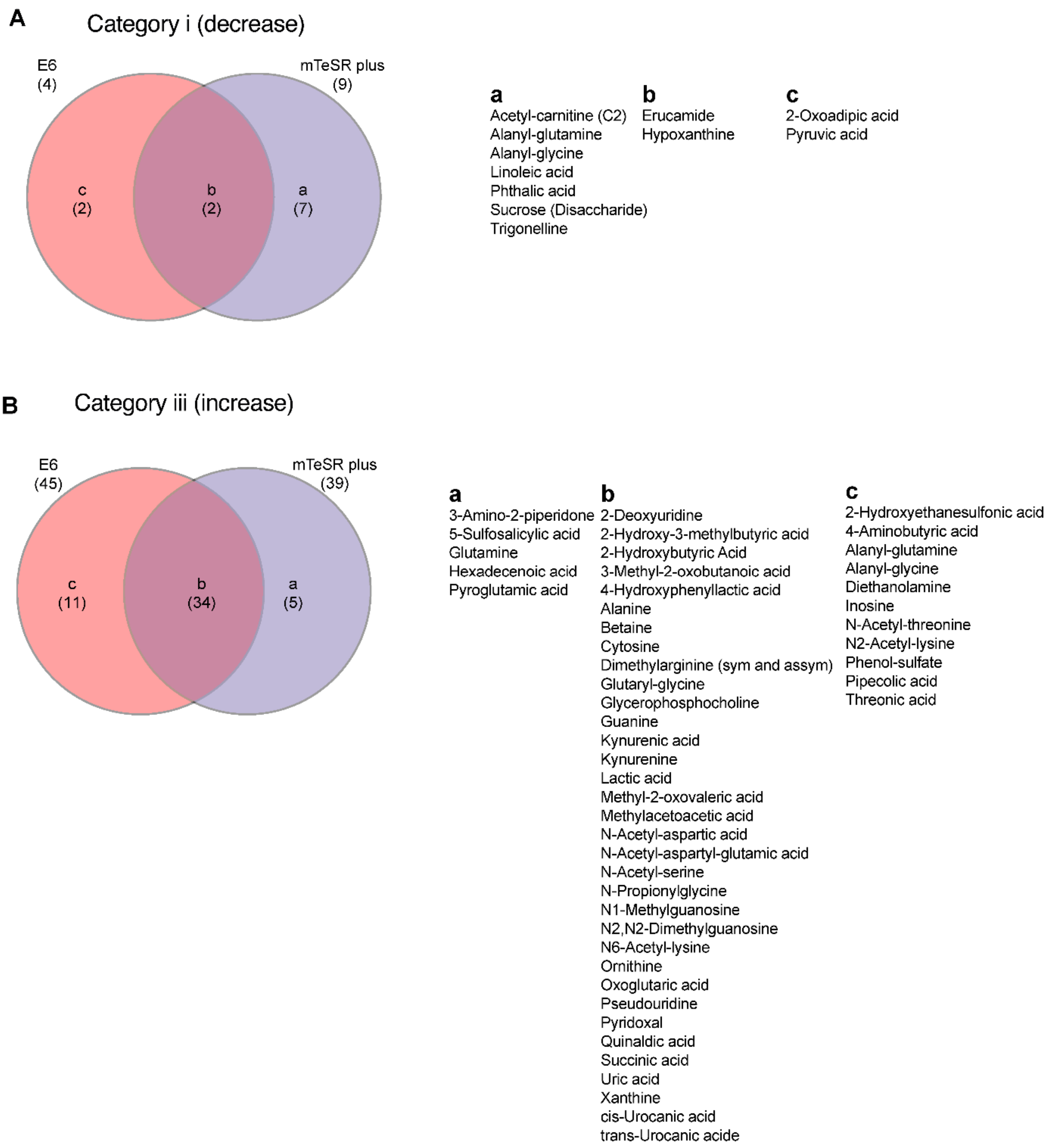

Figure S4. The determination of metabolites signatures under the differentiation conditions. Venn diagrams that highlight the differential shifts of metabolites between the hiPSC medium (mTeSR plus) and the differentiation medium E6. (**A**) In Category i (log2 fold change ≤ −1). (**B**) In Category iii (log2 fold change ≥ 1).
